# Early Initiation of Adalimumab Significantly Diminishes Postoperative Crohn’s Disease Endoscopic Recurrence and Is Superior to 6-Mercaptopurine Therapy: An Open-Label, Randomized Controlled Study

**DOI:** 10.3390/jcm12247600

**Published:** 2023-12-10

**Authors:** Ayal Hirsch, Erez Scapa, Naomi Fliss-Isakov, Hagit Tulchinsky, Eran Itzkowitz, Yehuda Kariv, Yulia Ron, Henit Yanai, Ian White, Sharief Yassin, Nathaniel Aviv Cohen, Eli Brazovski, Iris Dotan, Nitsan Maharshak

**Affiliations:** 1 Department of Gastroenterology and Liver Diseases, Tel Aviv Medical Center, Tel Aviv 6423906, Israel; ayalh@tlvmc.gov.il (A.H.); erezs@tlvmc.gov.il (E.S.); naomishfi@gmail.com (N.F.-I.); yuliar@tlvmc.gov.il (Y.R.); shariefyas@gmail.com (S.Y.); nathanielac@tlvmc.gov.il (N.A.C.); 2 Sackler Faculty of Medicine, Tel Aviv University, Tel Aviv 6423906, Israel; hagitt@tlvmc.gov.il (H.T.); erani@tlvmc.gov.il (E.I.); yehudaka@tlvmc.gov.il (Y.K.); henitya@clalit.org.i (H.Y.); ian@drianwhite.co.il (I.W.); ebraz@tlvmc.gov.il (E.B.); irisdo@clalit.org.il (I.D.); 3 Department of Surgery, Tel-Aviv Sourasky Medical Center, Tel Aviv 6423906, Israel; 4 Division of Gastroenterology, Rabin Medical Center, Petah-Tikva 4941492, Israel; 5 Department of General Surgery, Rabin Medical Center, Petah-Tikva 4941492, Israel; 6 The Pathology Department, Tel Aviv Medical Center, Tel Aviv 6423906, Israel

**Keywords:** clinical trial, Crohn, surgery, 6-mercaptopurine, adalimumab

## Abstract

Postoperative recurrence (POR) is the rule in patients with Crohn’s disease (CD), mitigated with prophylactic therapy. The evidence for therapeutic choice and timing of intervention is lacking. We aimed to compare the rates of POR in patients treated early with prophylactic 6-mercaptopurine (6-MP) or adalimumab. We conducted a prospective single-center randomized open-label clinical study in which patients in surgical remission following their first ileocecectomy were randomized to receive early treatment with 6-MP or adalimumab. Patients were followed up clinically every 3 months and underwent endoscopy at weeks 32 and 58 postoperatively. The primary endpoint was endoscopic recurrence (ePOR) at 1 year (week 58), defined as a Rutgeerts score ≥ i2. We enrolled 35 patients (25 males, mean age 35 ± 1.4 years, median disease duration 5 ± 6.1 years) following ileocecectomy. Of these, seven (20%) were current smokers and nine (26%) biologics-experienced. Patients allocated to adalimumab had significantly less ePOR than patients treated with 6MP at week 32 (21% vs. 69%, *p* = 0.004) and 58 (47% vs. 75%), (*p* = 0.03, HR = 0.39, 95% CI = 0.16–0.93). POR was associated with an increased diameter of the resected small bowel surgical specimen, lower baseline body mass index (BMI), increased week 18 fecal calprotectin, increased week 18 serum alanine aminotransferase and decreased week 18 hemoglobin level. Adalimumab was more effective than 6-MP in preventing ePOR. Increased operative small bowel diameter and lower postoperative BMI were associated with ePOR. At eighteen weeks, serum hemoglobin, ALT and fecal calprotectin levels were predictive of endoscopic disease recurrence. (ClinicalTrials.gov ID NCT01629628).

## 1. Introduction

Despite the advancements in medical therapy, a significant proportion of patients with Crohn’s disease (CD) undergo surgery, most commonly ileocolectomy, during their disease course [1,2]. Unfortunately, postoperative anastomotic recurrence (POR) is the rule, leading to additional surgery in up to a third of patients at 10 years [2,3,4]. Endoscopic postoperative recurrence (ePOR), assessed by the Rutgeerts score [5], precedes and predicts the clinical symptoms and progression to surgery [6]. 

Smoking, penetrating disease, submucosal myenteric plexitis, previous small bowel resections and histologic inflammation in the resected bowel margins were identified, among others, as risk factors for early disease recurrence [7,8,9,10]. The American Gastroenterological Association institute guidelines suggest postoperative early pharmacological prophylactic treatment with thiopurines or TNF-α inhibitors (TNFis) over endoscopic monitoring, started within 8 weeks of surgery [11]. The first surveillance colonoscopy is recommended 6–12 months following surgery. Clinical studies comparing prophylaxis with thiopurines and TNFis yielded conflicting evidence [12,13,14,15,16]. We aimed to compare rates of ePOR in patients treated early with prophylactic 6-mercaptopurine (6-MP) or adalimumab.

## 2. Materials and Methods

### 2.1. Study Design and Population

We conducted a prospective, single-center, randomized, open-label clinical study aimed to compare the efficacy of adalimumab to 6-MP for the maintenance of postoperative remission in patients with CD.

Patients with CD who underwent their first ileocecectomy due to active disease were screened 2 weeks postoperatively. Inclusion criteria: 18–70 years; clinical remission, i.e., Crohn’s disease activity index (CDAI) ≤ 150; eligible for treatment with adalimumab or 6MP. Exclusion criteria: residual inflammatory disease, i.e., residual ileal or colonic inflammation or perianal disease; experienced prior nonresponse or intolerance to TNFi agents; received antibiotics or mesalamine at baseline; had evidence of current infectious disease or severe or progressive systemic disease.

Eligible consecutive patients were allocated randomly in a 1:1 ratio to adalimumab or 6-MP treatment groups. Patients were randomized using a predetermined randomization log. Based on a predetermined 1:1 ratio, patients were assigned to either therapy in blocks of 10. Within each block, patients were assigned to each treatment arm in a randomized fashion.

### 2.2. Study Interventions

Treatment was started within 4 weeks following randomization. 6-MP was administered orally, starting at a dose of 50 mg/day with escalating doses every 1–2 weeks as tolerated to a target dose of 1–1.5 mg/kg. Adalimumab was administered as subcutaneous injections (SC) using prefilled syringes (40 mg/syringe). The dosing regimen was as follows. Induction: week 0—160 mg; week 2—80 mg; week 4—40 mg. Maintenance: 40mg every other week. No treatment optimizations were performed.

### 2.3. Follow-Up

Scheduled clinic visits were conducted at baseline and 18, 32, 42 and 58 weeks postsurgery thereafter. Pathological data, including maximal small bowel diameter and inflammation of margins, was extracted from the surgical pathology report at baseline. At each visit, patients were assessed for body weight and disease activity by calculating the CDAI score and quality of life using the Inflammatory Bowel Disease Questionnaire (IBDQ) and the SF-36 health survey. Clinical activity was defined using the CDAI score (<150 for clinical remission, 150–220 for mild clinical activity and >220 for moderately to severely active disease). Blood and stool samples were collected and analyzed for blood count, C-reactive protein and fecal calprotectin, respectively. Fecal calprotectin was assessed using the IDK^®^ Calprotectin ELISA test (Immundiagnostik AG, Stubenwald-Allee 8a, 64625 Bensheim, Germany) as recommended by the manufacturer. All patients were scheduled to undergo colonoscopies at weeks 32 and 58 postoperatively to assess for disease recurrence using the Rutgeerts endoscopic scoring system. A Rutgeerts score of ≥i2 was regarded as ePOR and was followed by a withdrawal from the study. Two physicians who were blinded to the patients’ study group performed the endoscopies and graded the anastomotic appearance. 

Treatment failure resulting in withdrawal from the study was defined in patients with CD exacerbation during a 1-year treatment period as an increase of ≥70 points in CDAI compared to baseline and a total CDAI score ≥ 220, Rutgeerts score ≥ i2, patients requiring re-operation or starting steroid therapy, patients experiencing significant hypersensitivity reactions (e.g., bronchospasm, anaphylaxis or urticaria) to study medications, or if the patient was not compliant with the study protocol. 

### 2.4. Study Outcomes

The primary endpoint was the proportion of patients with ePOR (Rutgeerts score ≥ i2) at 58 weeks postoperatively.

The study’s secondary endpoints were ePOR rate at 32 weeks, proportion of patients at clinical remission at 32 and 58 weeks, time to clinical relapse, mean serum C-reactive protein (CRP) levels, mean serum TNFi levels, proportion of patients experiencing adverse events, and quality of life scores (IBDQ and SF-36 health survey).

The study protocol was approved by the Institutional Review Board of the Tel Aviv Medical Center, and all participants provided informed consent prior to the study enrollment. The study was listed as a clinical trial with a ClinicalTrials.gov Identifier: NCT01629628.

### 2.5. Statistical Analysis

We aimed to recruit a total of 100 patients, with a 1:1 ratio between treatment arms. We assumed an endoscopic recurrence rate of 60% at 1 year on 6MP therapy and a 50% decrease in endoscopic recurrence on TNFi therapy. A total sample size of 100 patients was calculated to provide a power of 0.8 (α = 0.05). Unfortunately, patient enrollment did not meet expectations, partially due to the approval of additional CD therapies by the Israeli Ministry of Health, offering alternative postoperative therapeutic options.

Descriptive statistics were calculated for each variable measured and are reported as means, medians or proportions. Univariate analyses for differences in patients’ characteristics and demographics were determined using paired *t*-tests or Wilcoxon rank-sum tests for means and McNemar tests for categorical variables. A *p*-value < 0.05 was considered significant. Kaplan-Meier estimate curves were plotted for relapse-free survival. Hazard ratios (HRs) for relapse-free survival were estimated with Cox regression and, stratified by treatment arm and variable, were found to be statistically significant for predicting POR.

## 3. Results

Forty-one patients with CD who underwent a first ileocolonic surgical resection for active CD during 2012–2018 were screened. The study was terminated by the PIs after an enrollment of 41% of the intended cohort due to futility in recruitment rate. Patients were excluded for early postoperative complications (*n* = 1), CD involving proximal small bowel (*n* = 1) and withdrawal of consent (*n* = 4), leaving 35 study-eligible patients, 25 males, mean age 35 ± 1.4 years, randomized to receive either 6-MP (*n* = 16) or adalimumab (*n* = 19). Study arms were comparable in baseline demographic parameters, BMI, smoking status, clinical scores and surgical findings, as seen in Table 1. Preoperative therapy was also comparable between the study groups. Specifically, 11 (31%) patients were treated with TNFis preoperatively; of these, 7 (64%) were treated with adalimumab during the study, and 4 (36%) were started on 6-MP.

### 3.1. Study Timeline

The actual study timeline was similar to the timeline projected in the protocol. Following surgery, patients were screened for the study at 4 (median, range: 1.7–8.9) weeks, commenced therapy with the allocated treatment at 6.4 (median, range: 2.6–15.2) weeks and underwent surveillance endoscopies at 32.9 (median, range:25.6–42) weeks and 58.4 (median, range: 51.6–86) weeks.

### 3.2. ePOR at Week 32

All 35 patients underwent ileocolonoscopy 32.9 weeks following surgery. Of these, 15 (43%) patients were determined to have ePOR (Rutgeerts ≥ i2).

**Figure 1:**
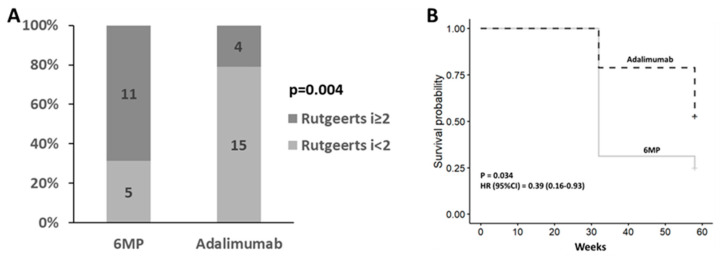
Week 32 postoperative endoscopic recurrence by study arm (**A**), Kaplan-Meier curve of postoperative Crohn’s disease recurrence-free survival by study arm (**B**).

Postoperative treatment with adalimumab was associated with a lower recurrence rate compared with 6-MP therapy (4[21%] vs. 11[69%], *p* = 0.004) with a relative risk of 0.3 (95% CI: 0.12–0.78) (Figure 1A). Patients treated with anti-TNF had lower recurrence rates compared with 6MP, regardless of smoking status (14% vs. 72% and 25% vs. 66% for smokers and nonsmokers, respectively).

### 3.3. Endoscopic Recurrence at Week 58

In the intention-to-treat cohort, 20 (63%) patients experienced ePOR at week 58. Of these, 12 (75%) were in the 6MP study arm and 9 (47%) in the adalimumab arm. Postoperative treatment with adalimumab was associated with a significantly lower recurrence rate compared with 6-MP (*p* = 0.03, HR = 0.39, 95% CI = 0.16–0.93) (Figure 1B).

### 3.4. Factors Associated with Endoscopic POR

#### 3.4.1. Small Bowel Diameter

**Figure 2:**
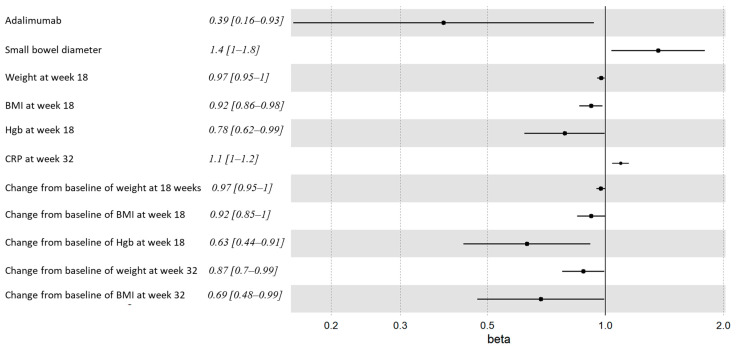
Forest plot of week 58 surgical recurrence hazard ratios (HR [95%CI]).

Increased resected small bowel diameter, as measured in the pathology lab, was associated with ePOR at weeks 32 and 58 (3.5 ± 1.8 cm vs. 3.1 ± 0.8 cm, *p* = 0.05) and (*p* = 0.023, HR = 1.4, 95% CI = 1–1.8) (Table 2, Appendix A Table A1 and Figure 2).

#### 3.4.2. Inflammatory Indices

**Figure 3:**
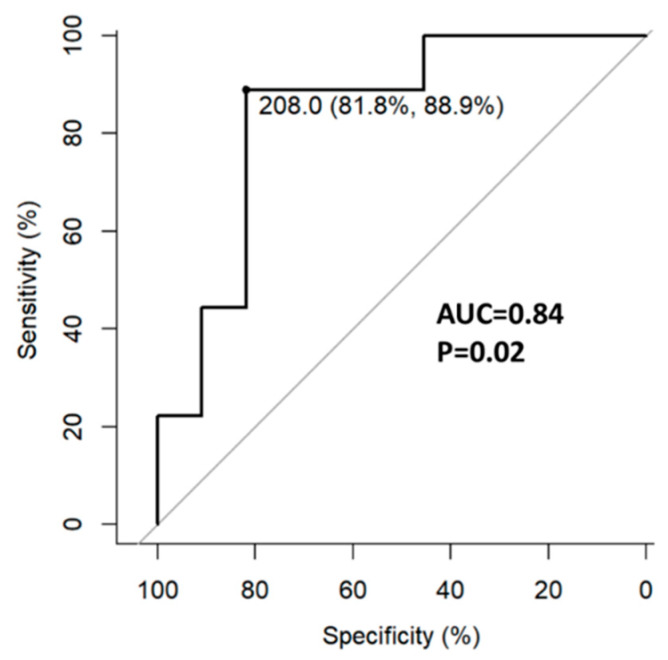
Fecal calprotectin concentration at week 18 predicts week 32 endoscopic recurrence.

All 35 patients had week 18 and 58 labs. Higher week 18 fecal calprotectin concentration was associated with week 32 ePOR. The optimal cut-off point associated with ePOR was 208 µg/gr (AUC = 0.84, 95%CI 0.65–1.0) with a sensitivity of 89% and specificity of 82% (Figure 3).

Higher week 32 CRP levels were associated with week 58 CD recurrence (*p* = 0.019, HR = 1.1, 95%CI = 1–1.2).

#### 3.4.3. Weight and BMI

**Figure 4:**
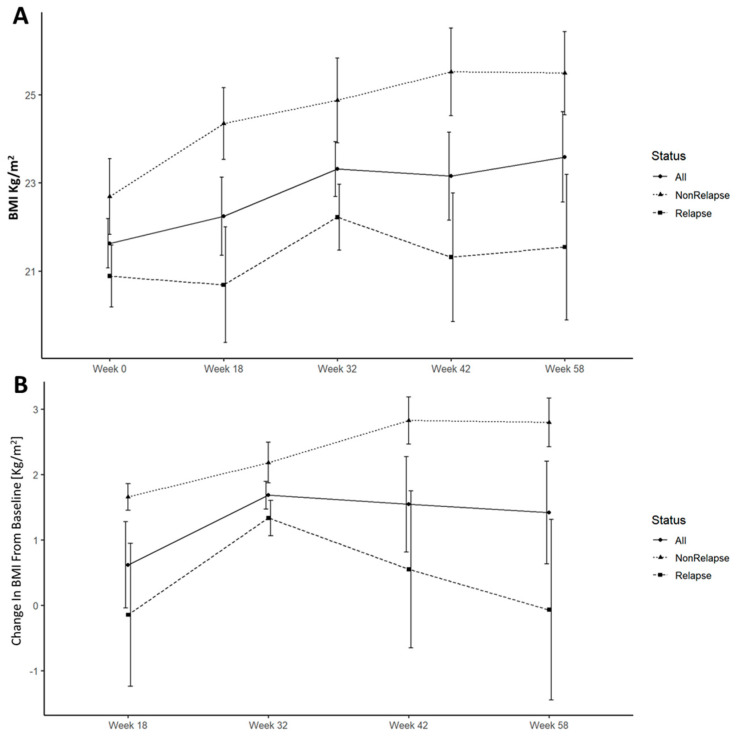
BMI (**A**) and change in BMI from baseline (**B**) by week 58 endoscopic recurrence (Rutgeerts score > i2).

Lower weight and BMI were associated with an increased risk of ePOR. This was noticed throughout the study duration and became statistically significant at weeks 18 and 32. (Figure 2 and Figure 4A). All patients gained weight and increased in BMI following surgery; however, this trend was significantly accentuated and durable in patients who remained disease-free at week 58 (Figure 4B).

#### 3.4.4. Hemoglobin

Lower absolute hemoglobin (Hgb) levels at week 18 and a decrease in Hgb levels at week 18 compared to baseline were associated with ePOR at weeks 32 and 58 (Table 3 and Appendix A Table A2, Figure 2).

#### 3.4.5. Alanine Aminotransferase

Higher alanine aminotransferase (ALT) levels at week 18 were associated with week 32 ePOR.

The associations of ALT and Hgb with ePOR remained even after excluding the 6-MP arm. Other baseline variables previously described as impactful, such as disease extent, smoking status or inflammation of the resected bowel margins, were not associated with disease recurrence in our patient cohort (Table 2).

### 3.5. Clinical Recurrence

Clinical recurrence occurred in three patients, and clinical scores and quality of life questionnaires were comparable for both study arms at 32 and 58 weeks (Table 3). Two patients progressed from clinical remission to mildly active clinical disease, and one patient (adalimumab arm) progressed from mildly active disease to severely active clinical disease.

### 3.6. Safety

Adverse events and severe adverse event rates were comparable in the two study arms. Overall, eight patients in the adalimumab arm reported 11 adverse events (examination with seton placement, neuropathy, rash, respiratory infections, folliculitis, psoriasis, alopecia) and 2 severe adverse events (abdominal pain, acute gastroenteritis) compared to the 6-MP arm where nine patients reported 13 adverse events (leukopenia, hepatitis, respiratory infections) and 6 severe adverse events (leukopenia, hepatitis, dermatitis, abdominal wall infection, pneumonia, iatrogenic colon perforation).

No drug intolerance or infusion reactions leading to drug or study withdrawal were reported.

## 4. Discussion

In this study, Postoperative Adalimumab Recurrence Trial (POPART), we aimed to compare the rates of POR in patients treated early with prophylactic 6-mercaptopurine (6-MP) or adalimumab. Recruiting 35 patients and following them clinically and using laboratory and endoscopic biomarkers, we demonstrated adalimumab superiority over 6-MP in preventing POR. We identified small bowel dilatation of the surgical specimen and lower postoperative weight/BMI as risk factors for ePOR. In addition, we identified several predictors of ePOR, including the rate of weight/BMI gain, week 18 serum Hgb, ALT and fecal calprotectin levels and week 32 CRP level.

International society guidelines recommend thiopurines and TNFis for prophylaxis of POR, regardless of patient risk stratification [17]. The recommendation is without preference for drug mechanism and infers equivalence in efficacy. Prospective, randomized studies comparing thiopurines and TNFis as a postoperative prophylaxis strategy are scarce, with inconsistent results.

In a pilot study, Armuzzi et al. compared 1-year ePOR in 22 patients at high risk for POR randomized for treatment with infliximab or azathioprine. Infliximab was numerically superior to azathioprine; however, the study was underpowered and did not achieve statistical significance. Savarino et al. performed a controlled, nonblinded study with 51 patients randomly allocated to three study arms comparing adalimumab, azathioprine and mesalamine [16]. The study included high-risk patients (smokers with previous small bowel resection) and looked at 1-year endoscopic and clinical recurrence. Surprisingly, only 6.3% (one patient) of the adalimumab-treated patients were found with ePOR compared with 64.7% and 83.3% in the azathioprine and mesalamine arms, respectively. Adalimumab was also significantly better at the prevention of clinical recurrence, with a 12.5% recurrence rate, compared with 64.7% and 50% in the azathioprine and mesalamine groups. The GETECCU study, by Lopez-Sanroman et al., was a randomized, controlled, open-label study comparing adalimumab and azathioprine in 61 patients, of which approximately 60% were at high risk for POR. All patients were treated with metronidazole for 3 months. ePOR rates at 58 weeks were comparable between adalimumab and azathioprine (29.7% vs. 33.3%, *p* = 0.76). A subanalysis of the POCER study compared adalimumab and azathioprine in high-risk patients, also treated with metronidazole for 3 months and scoped for ePOR at 6 months [14]. Adalimumab was superior to azathioprine in the prevention of POR (45% vs. 21%, *p* = 0.028). While the POCER study compared endoscopic-driven management strategies, we aimed to compare treatment efficacy. The difference in study goals manifested in a different design and study populations [12]. All the patients in the POCER study were treated with antibiotics, and only high-risk patients received early postoperative medical prophylaxis. Azathioprine was the default treatment for high-risk patients, and only thiopurine-intolerant patients were treated with adalimumab. We avoided initial antibiotic therapy in the POPART study to avoid a short-term reduction in POR, which may interfere with a direct comparison of the efficacy of 6MP and adalimumab at 6 and 12 months. In addition, in contrast to the patient risk stratification in POCER, all patients in the POPART study were randomly allocated to early treatment with 6MP or adalimumab. Furthermore, approximately 60% of the patients in the POCER study had preoperative bowel perforation, 40% were smokers, and 30–43% underwent prior surgeries. In comparison, the POPART study population had a milder disease course, no prior surgeries, and only 20% were smokers. Many of these patients would not have been eligible for early medical prophylaxis if they were enrolled in POCER. In short, in contrast to the POCER study, we randomly allocated mixed high- and low-risk populations, without stratification and initial antibiotic therapy, to early prophylactic treatment with 6MP or adalimumab. We found adalimumab was more effective than 6-MP in the prevention of POR at 32 and 58 weeks postoperatively. We were also able to show that disease recurrence was associated with increased resected small bowel diameter, which may be a marker of prestenotic dilatation. However, patients who suffered from fibrostenotic disease without an active inflammatory disease were not included in our study. 

Regarding biomarkers, increased 6-month fecal calprotectin was associated with ePOR in the POCER study [18]. Subsequent studies showed a 3-month fecal calprotectin level to be predictive of endoscopic recurrence [19,20]. Our results replicate these findings with fecal calprotectin ≥ 218 µg/gr having a sensitivity of 88% and specificity of 82% for disease recurrence. Another finding was the association of lower initial postoperative weight and BMI with endoscopic recurrence. In addition, ePOR was associated with lower weight/BMI increase during this study. These findings may be interpreted either as evidence of worse surgical outcomes resulting from poor presurgical dietary status or as persistent subacute inflammation in a more refractory patient population leading to initial and consequent lower weight and BMI. 

Furthermore, we found that at 3 months, decreased Hgb and increased ALT levels were predictive of ePOR. Decreased Hgb levels may represent a subclinical inflammation with subsequent iron loss and diminished absorption. Both increased ALT and decreased Hgb levels remained predictive of recurrence, even when excluding the 6-MP arm, precluding 6-MP shunting as a cause of this finding. The increase in ALT levels may be related to fatty changes of the liver occurring during the study follow-up, as an increase in BMI was also associated with a decrease in ePOR.

We found no association between clinical and endoscopic recurrence and overall showed very low clinical recurrence rates. These findings are in line with previous studies showing the disconnection between clinical and endoscopic findings and the lag of clinical following endoscopic recurrence, lending support to the choice of endoscopic recurrence as a monitoring metric in POR [6]. A longer study follow-up may have shown higher clinical recurrence rates.

Our study has merit, including its prospective study design and random allocation to study treatment. This study was performed in a single tertiary medical center, and patients were screened, enrolled and treated in a similar fashion. Two physicians performed and graded all the endoscopies, adding to the study procedures’ uniformity. We enrolled only patients undergoing ileocecectomy as their first surgical intervention and treated them without risk stratification or antibiotic treatment. This design not only offers a direct comparison of the effects of prophylaxis but is also streamlined and closer to real-world practice, avoiding complex patient stratification and biomarker monitoring with early therapy commencement.

However, our study is not without limitations, including the study’s open-label design, introducing patient and physician bias in evaluating clinical and endoscopic disease activity. We did, however, blind the physician who performed the endoscopies and graded the anastomoses. An additional weakness is a low patient recruitment rate, probably due to the introduction of novel advanced therapies and due to the single-center design, which may limit the generalizability of our findings. This led to the study’s termination before achieving the required number of patients. We were also unable to test for serum thiopurine metabolites and serum adalimumab levels, which means that our therapy was not optimized proactively, possibly underutilizing our medical options and increasing recurrence rates.

## 5. Conclusions

In conclusion, we show that adalimumab is more effective than 6-MP in postoperative CD prophylaxis. Increased surgical operative small bowel diameter and lower postoperative weight/BMI were associated with ePOR. Three-month serum Hgb and ALT levels and stool calprotectin concentrations are predictive of ePOR. Our findings may improve the treatment of postoperative CD patients, especially in the low-risk patient group, and assist clinicians in the choice of pharmacologic prophylaxis.

## Figures and Tables

**Table 1:** Baseline Characteristics.

Study Population (*n* = 35)	6-MP (*n* = 16)	Adalimumab (*n* = 19)	*p*-Value
Age [years] [mean ± SE]	31.3 ± 1.9	33.1 ± 2.1	0.5
Males *n* (%)	11 (69)	14 (74)	1
Disease duration [years] [median ± SD]	2.5 ± 6.7	5 ± 5.7	0.9
Smoking *n* (%)			
Never/Past	11 (69)	17 (89)	0.3
Current	5 (31)	2 (11)	
Preop therapy *n* (%)			
6MP	7 (44)	4 (21)	0.16
Anti-TNF	2 (13)	7 (36)	
Duration: surgery to therapy [days] [median ± SD]	44 ± 18.8	46.7 ± 6.4	1
Ethnicity *n* (%)			
Ashkenazi	6 (38)	7 (36)	1
Non-Ashkenazi	8 (50)	10 (53)	
BMI [Kg/m^2^] [mean ± SE]	20.3 ± 0.7	22.3 ± 0.9	0.1
Lab at baseline			
Hemoglobin [g/dL] [mean ± SE]	12.5 ± 0.5	12.3 ± 0.4	0.7
WBC [10 × 10^3^/µL] [median ± SD]	6.9 ± 2.5	6.75 ± 2	1
PLT [10 × 10^3^/µL] [median ± SD]	275 ± 102.7	282 ± 178.2	0.6
ALT [U/L] [mean ± SE]	32 ± 33	39.5 ± 35.7	0.4
AST [U/L] [median ± SD]	23 ± 19	32 ± 12.4	0.6
CRP [mg/dL] [median ± SD]	1.8 ± 2.8	4.7 ± 6.8	0.1
Stool calprotectin [µg/gr] [median ± SD]	149 ± 316.1	234 ± 203	0.9
Clinical scores			
IBDQ [mean ± SE]	183.9 ± 6.5	171.3 ± 6.8	0.2
SF36 [mean ± SE]	2484 ± 139.9	2075 ± 148.5	0.06
CDAI [median ± SD]	112 ± 41.1	112.4 ± 45.9	0.9
Surgical variables			
Small bowel resected length [cm] [median ± SD]	12.5 ± 17	16 ± 7.6	0.8
Small bowel diameter [cm] [median ± SD]	3.5 ± 1.7	3.5 ± 1	0.1
Colon resected length [cm] [median ± SD]	8 ± 12	7 ± 2.3	0.1
Appendiceal inflammation, *n* (%)	6 (50)	4 (29)	0.5
Proximal margin inflammation, *n* (%)	4 (29)	2 (12)	0.5
Distal margin inflammation, *n* (%)	2 (14)	1 (6)	0.9

Abbreviations: 6MP (6-mercaptopurine), BMI (Body Mass Index), WBC (White Blood Cells), PLT (Platelets), ALT (Alanine aminotransferase), AST (Aspartate aminotransferase), CRP (C-reactive protein), IBDQ (Inflammatory Bowel Disease Questionnaire), SF36 (36-Item Short Form Survey), CDAI (Crohn’s Disease Activity Index), SE (Standard Error), SD (Standard Deviation).

**Table 2:** Week 32 endoscopic recurrence by baseline characteristics.

Study Population (*n* = 35)	Rutgeerts < i2 (*n* = 20)	Rutgeerts ≥ i2 (*n* = 15)	*p*-Value
Study drug *n* (%)			
6 MP	5 (25)	11 (73)	0.004
adalimumab	15 (75)	4 (27)	
Age [years] [mean ± SE]	33.2 ± 1.9	31 ± 2	0.4
Males *n* (%)	13 (65)	12 (80)	0.6
Disease duration [years] [median ± SD]	5 ± 6.1	2.5 ± 6.2	0.6
Smoking *n* (%)			
Never/Past	18 (90)	10 (66)	0.2
Current	2 (10)	5 (34)	
Duration: surgery to therapy [days] [median+SD]	46 ± 16.2	45 ± 8.1	0.4
BMI [Kg^2^/m] [median ± SE] at baSEline	22 ± 0.8	20.5 ± 0.9	0.2
Labs at baseline			
Hemoglobin [g/dL] [mean ± SE]	12.2 ± 0.3	12.6 ± 0.5	0.6
WBC [10 × 10^3^/µL] [median ± SD]	6.5 ± 1.6	7.5 ± 2.7	0.1
PLT [10 × 10^3^/µL] [median ± SD]	299 ± 126	262.5 ± 176.5	0.8
ALT [U/L] [median+SD]	32 ± 30	38 ± 40.2	0.6
AST [U/L] [median ± SD]	27 ± 13.9	29.5 ± 18.1	0.7
CRP [mg/dL] [median ± SD]	2.4 ± 5	5.2 ± 7	0.5
Stool calprotectin [µg/gr] [mean ± SE]	169 ± 207	202 ± 299.5	0.3
Clinical scores at baseline			
IBDQ [mean ± SE]	176 ± 6.5	179 ± 7.2	0.7
SF36 [mean ± SE]	2273 ± 145	2247 ± 165	0.9
CDAI [median ± SD]	102 ± 47.4	119 ± 36.3	0.3
Surgical variables			
Small bowel resected [cm] [median ± SD]	15.75 ± 7.4	15 ± 17.5	0.4
Small bowel diameter [cm] [median ± SD]	3.1 ± 0.8	3.5 ± 1.8	0.05
Colon resected [cm] [median ± SD]	7 ± 2.8	7.5 ± 12.5	0.1
Appendiceal inflammation *n* (%)	6 (40)	4 (36)	1
Resected margin inflammation *n* (%)	4 (20)	5 (33)	0.4

Abbreviations: BMI (Body Mass Index), WBC (White Blood Cells), PLT (Platelets), ALT (Alanine aminotransferase), AST (Aspartate aminotransferase), CRP (C-reactive protein), IBDQ (Inflammatory Bowel Disease Questionnaire), SF36 (36-Item Short Form Survey), CDAI (Crohn’s Disease Activity Index), SE (Standard Error), SD (Standard Deviation).

**Table 3:** Week 32 endoscopic recurrence (Rutgeerts ≥ i2) by week 18 parameters.

Study Population (*n* = 35)	Rutgeerts < i2 (*n* = 20)	Rutgeerts ≥ i2 (*n* = 15)	*p*-Value
BMI [Kg/m^2^] [median ± SD] at week 18	22.8 ± 3.6	20.5 ± 6.7	0.1
Labs at week 18			
Hemoglobin [g%] [mean ± SE]	13.6 ± 0.4	12.1 ± 0.5	0.03
WBC [10 × 10^3^/µL] [mean ± SE]	6 ± 0.3	5.8 ± 0.6	0.7
PLT [10 × 10^3^/µL] [median ± SD]	256 ± 69	262 ± 77	0.3
ALT [U/L] [median ± SD]	26 ± 18	19.5 ± 8.2	0.02
AST [U/L] [median ± SD]	23 ± 9.9	19 ± 5.8	0.09
CRP [mg/dL] [median ± SD]	0.6 ± 1.7	2.6 ± 14.2	0.07
Stool calprotectin [µg/gr] [median ± SD]	102 ± 145	303 ± 267	0.02
Clinical scores at week 18			
IBDQ [median ± SD]	190 ± 29.2	193 ± 33	0.6
SF36 [mean ± SE]	2593 ± 142	2382 ± 189	0.4
CDAI [mean ± SE]	76.4 ± 14.3	103.7 ± 16	0.2

Abbreviations: BMI (Body Mass Index), WBC (White Blood Cells), PLT (Platelets), ALT (Alanine aminotransferase), AST (Aspartate aminotransferase), CRP (C-reactive protein), IBDQ (Inflammatory Bowel Disease Questionnaire), SF36 (36-Item Short Form Survey), CDAI (Crohn’s Disease Activity Index), SE (Standard Error), SD (Standard Deviation).

## Data Availability

The data underlying this article will be shared on reasonable request to the corresponding author.

## Appendix A

**Table A1:** Hazard ratio for endoscopic recurrence at week 58 postsurgery.

Study Population (*n* = 35)	Rutgeerts < i2 (*n* = 14)	Rutgeerts ≥ i2 (*n* = 21)	HR (95% CI)
Study drug *n* (%)			
6 MP	4 (25)	12 (75)	0.39 (0.16–0.93)
adalimumab	10 (53)	9 (47)	
Small bowel diameter [cm] [median ± SD]	3.25 ± 0.9	3.5 ± 1.6	1.4 (1–1.8)
Week 18			
Weight [Kg] [median ± SD]	71 ± 13.8	61 ± 19.1	0.97 (0.95–1)
BMI [Kg/m^2^] [median ± SD]	24 ± 3	20.5 ± 6	0.92 (0.86–0.98)
Hemoglobin [g/dL] [mean ± SE]	13.7 ± 0.5	12.5 ± 0.4	0.78 (0.62–0.99)
Week 32			
CRP [mg/dL] [median ± SD]	1.5 ± 1.9	2.1 ± 6	1.1 (1–1.2)
Change from baseline at week 18			
Weight [Kg] [median ± SD]	4.6 ± 2.5	2.8 ± 15.5	0.97 (0.95–1)
BMI [Kg^2^/m] [median ± SD]	1.7 ± 0.8	1 ± 5	0.92 (0.85–1)
Hemoglobin [g/dL] [mean ± SE]	1 ± 0.3	0.16 ± 0.3	0.63 (0.44–0.91)
Change from baseline at week 32			
Weight [Kg] [mean ± SE]	6.7 ± 1	3.6 ± 0.8	0.87 (0.77–0.99)
BMI [Kg^2^/m] [mean ± SE]	2.2 ± 0.3	1.3 ± 0.3	0.69 (0.48–0.99)
Change from week 18 at week 32			
Hemoglobin [g/dL] [mean ± SE]	0.17 ± 0.2	1 ± 0.2	1.8 (1.2–2.6)

Abbreviations: BMI (Body Mass Index), WBC (White Blood Cells), PLT (Platelets), ALT (Alanine aminotransferase), AST (Aspartate aminotransferase), CRP (C-reactive protein), IBDQ (Inflammatory Bowel Disease Questionnaire), SF36 (36-Item Short Form Survey), CDAI (Crohn’s Disease Activity Index), SE (Standard Error), SD (Standard Deviation).

**Table A2:** Week 32 endoscopic recurrence by week 18 characteristics changes from baseline.

Study Population (*n* = 35)	Rutgeerts < i2 (*n* = 20)	Rutgeerts ≥ i2 (*n* = 15)	*p*-Value
BMI delta from baseline [Kg/m^2^] [median ± SD]	1.7 ± 1	0.9 ± 6	0.1
Labs (change from baseline at week 18)			
Hemoglobin [g/dL] [mean ± SE]	1.1 ± 0.3	−0.4 ± 0.3	0.0005
WBC [10 × 10^3^/µL] [median ± SD]	−0.55 ± 2	−1.1 ± 3.3	0.5
PLT [10 × 10^3^/µL] [median ± SD]	−20 ± 67	−7.5 ± 31	0.3
ALT [U/L] [mean ± SE]	-5 ± 4	−12 ± 3.4	0.2
AST [U/L] [median ± SD]	−1 ± 9.4	−7.5 ± 7.3	0.3
CRP [mg/dL] [median ± SD]	−2.42 ± 4.2	−0.3 ± 18.8	0.6
Stool calprotectin [µg/gr] [mean ± SE]	−155.3 ± 24.2	−18.8 ± 83	0.4
Clinical scores delta from baseline at week 18			
IBDQ [mean ± SE]	6.4 ± 4.4	2.6 ± 6.8	0.7
SF36 [mean ± SE]	367 ± 137	192 ± 137	0.4
CDAI [median ± SD]	−14 ± 42.3	−10 ± 59.2	0.5

Abbreviations: BMI (Body Mass Index), WBC (White Blood Cells), PLT (Platelets), ALT (Alanine aminotransferase), AST (Aspartate aminotransferase), CRP (C-reactive protein), IBDQ (Inflammatory Bowel Disease Questionnaire), SF36 (36-Item Short Form Survey), CDAI (Crohn’s Disease Activity Index), SE (Standard Error), SD (Standard Deviation).

## References

[1] Dittrich A.E., Sutton R.T., Haynes K., Wang H., Fedorak R.N., Kroeker K.I. (2020). Incidence Rates for Surgery in Crohn’s Disease Have Decreased: A Population-based Time-trend Analysis. Inflamm. Bowel Dis..

[2] Stoss C., Stöss C., Berlet M., Reischl S., Nitsche U., Weber M.C., Friess H., Wilhelm D., Neumann P.A. (2021). Crohn’s disease: A population-based study of surgery in the age of biological therapy. Int. J. Colorectal. Dis..

[3] Peyrin-Biroulet L., Harmsen S.W., Tremaine W.J., Zinsmeister A.R., Sandborn W.J., Loftus E.V. (2012). Surgery in a population-based cohort of Crohn’s disease from Olmsted County, Minnesota (1970–2004). Am. J. Gastroenterol..

[4] Szanto K., Nyári T., Balint A., Bor R., Milassin Á., Rutka M., Fábián A., Szepes Z., Nagy F., Molnar T. (2018). Biological therapy and surgery rates in inflammatory bowel diseases—Data analysis of almost 1000 patients from a Hungarian tertiary IBD center. PLoS ONE.

[5] Rutgeerts P., Geboes K., Vantrappen G., Beyls J., Kerremans R., Hiele M. (1990). Predictability of the postoperative course of Crohn’s disease. Gastroenterology.

[6] Ble A., Renzulli C., Cenci F., Grimaldi M., Barone M., Sedano R., Chang J., Nguyen T.M., Hogan M., Zou G. (2022). The Relationship Between Endoscopic and Clinical Recurrence in Postoperative Crohn’s Disease: A Systematic Review and Meta-analysis. J. Crohn’s Colitis.

[7] Cottone M., Rosselli M., Orlando A., Oliva L., Puleo A., Cappello M., Traina M., Tonelli F., Pagliaro L. (1994). Smoking habits and recurrence in Crohn’s disease. Gastroenterology.

[8] McLeod R.S., Wolff B.G., Ross S., Parkes R., McKenzie M. (2009). Recurrence of Crohn’s disease after ileocolic resection is not affected by anastomotic type: Results of a multicenter, randomized, controlled trial. Dis. Colon Rectum.

[9] Avidan B., Sakhnini E., Lahat A., Lang A., Koler M., Zmora O., Bar-Meir S., Chowers Y. (2005). Risk factors regarding the need for a second operation in patients with Crohn’s disease. Digestion.

[10] Pascua M., Su C., Lewis J.D., Brensinger C., Lichtenstein G.R. (2008). Meta-analysis: Factors predicting post-operative recurrence with placebo therapy in patients with Crohn’s disease. Aliment. Pharmacol. Ther..

[11] Adamina M., Bonovas S., Raine T., Spinelli A., Warusavitarne J., Armuzzi A., Bachmann O., Bager P., Biancone L., Bokemeyer B. (2020). ECCO Guidelines on Therapeutics in Crohn’s Disease: Surgical Treatment. J. Crohn’s Colitis.

[12] De Cruz P., Kamm M.A., Hamilton A.L., Ritchie K.J., Krejany E.O., Gorelik A., Liew D., Prideaux L., Lawrance I.C., Andrews J.M. (2015). Crohn’s disease management after intestinal resection: A randomised trial. Lancet.

[13] Armuzzi A., Felice C., Papa A., Marzo M., Pugliese D., Andrisani G., Federico F., De Vitis I., Rapaccini G.L., Guidi L. (2013). Prevention of postoperative recurrence with azathioprine or infliximab in patients with Crohn’s disease: An open-label pilot study. J. Crohn’s Colitis.

[14] De Cruz P., Kamm M.A., Hamilton A.L., Ritchie K.J., Krejany E.O., Gorelik A., Liew D., Prideaux L., Lawrance I.C., Andrews J.M. (2015). Efficacy of thiopurines and adalimumab in preventing Crohn’s disease recurrence in high-risk patients—A POCER study analysis. Aliment. Pharmacol. Ther..

[15] Lopez-Sanroman A., Vera-Mendoza I., Domènech E., Taxonera C., Vega Ruiz V., Marín-Jiménez I., Guardiola J., Castro L., Esteve M., Iglesias E. (2017). Adalimumab vs Azathioprine in the Prevention of Postoperative Crohn’s Disease Recurrence. A GETECCU Randomised Trial. J. Crohn’s Colitis.

[16] Savarino E., Bodini G., Dulbecco P., Assandri L., Bruzzone L., Mazza F., Frigo A.C., Fazio V., Marabotto E., Savarino V. (2013). Adalimumab is more effective than azathioprine and mesalamine at preventing postoperative recurrence of Crohn’s disease: A randomized controlled trial. Am. J. Gastroenterol..

[17] Nguyen G.C., Loftus E.V., Hirano I., Falck–Ytter Y., Singh S., Sultan S., Flamm S.L., Lim J.K., Rubenstein J.H., Smalley W.E. (2017). American Gastroenterological Association Institute Guideline on the Management of Crohn’s Disease After Surgical Resection. Gastroenterology.

[18] Wright E.K., Kamm M.A., De Cruz P., Hamilton A.L., Ritchie K.J., Krejany E.O., Leach S., Gorelik A., Liew D., Prideaux L. (2015). Measurement of fecal calprotectin improves monitoring and detection of recurrence of Crohn’s disease after surgery. Gastroenterology.

[19] Boube M., Laharie D., Nancey S., Hebuterne X., Fumery M., Pariente B., Roblin X., Peyrin-Biroulet L., Minet-Quinard R., Pereira B. (2020). Variation of faecal calprotectin level within the first three months after bowel resection is predictive of endoscopic postoperative recurrence in Crohn’s disease. Dig. Liver Dis..

[20] Veyre F., Boschetti G., Meunier C., Cuerq C., Gay C., Charlois A.L., Duclaux-Loras R., Danion P., Cotte E., Kepenekian V. (2021). Low Levels of Fecal Calprotectin 3 Months After Surgery Predict Subsequent Endoscopic Postoperative Remission in Crohn’s Disease. Dig. Dis. Sci..

